# Efficacy and Safety of Vancomycin, Linezolid, and Ceftaroline in the Treatment of Methicillin-Resistant Staphylococcus aureus (MRSA): A Systematic Review and Meta-Analysis

**DOI:** 10.7759/cureus.77949

**Published:** 2025-01-25

**Authors:** Ibrahim M Dighriri, Sarah Alanazi, Khalid AlMutairi, Sarah J Alhusayni, Fatimah M Balharith, Reem A Aljuwaie, Hailah K Alfayez, Ghadi M Althubaiti, Ghada A Alosaimi, Osama W Jameel, Asalah M Alansari

**Affiliations:** 1 Department of Pharmacy, King Abdulaziz Specialist Hospital, Taif, SAU; 2 Department of Pharmacy, King Khalid Hospital, Najran, SAU; 3 Department of Pharmacy, Dr. Sulaiman Al Habib Hospital, Riyadh, SAU; 4 Department of Pharmacy, Nahdi Pharmacy, Medina, SAU; 5 Department of Pharmacy, East Jeddah Hospital, Jeddah, SAU; 6 Department of Pharmacy, Specialized Medical Center Hospital, Riyadh, SAU; 7 College of Pharmacy, Princess Nourah Bint Abdulrahman University, Riyadh, SAU; 8 Department of Pharmacy, Kol Alosra Pharmacy, Taif, SAU; 9 Department of Pharmacy, United Pharmacy, Taif, SAU; 10 Department of Pharmacy, Nahdi Pharmacy, Riyadh, SAU; 11 College of Pharmacy, Taif University, Taif, SAU

**Keywords:** ceftaroline, linezolid, mortality rates, mrsa infections, thrombocytopenia, vancomycin

## Abstract

Methicillin-resistant *Staphylococcus aureus* (MRSA) is one of the world's most serious healthcare issues, causing morbidity, mortality, and high healthcare costs, making effective treatment strategies essential. This meta-analysis assessed the comparative effectiveness and safety of ceftaroline, linezolid, and vancomycin in treating MRSA infections. Searches were undertaken across major electronic databases, including PubMed, Cochrane CENTRAL, Embase, and Web of Science, from 2000 to 2024. A total of 24 studies (17 randomized controlled trials (RCTs) and seven observational studies) involving 11,332 patients met the inclusion criteria. Patients on vancomycin treatment were more likely to have lower odds of being cured than controls (OR 0.68; 95% CI (0.58, 0.81), p < 0.0001), especially when compared to linezolid (OR 0.61; 95% CI (0.49, 0.74), p < 0.00001). Furthermore, mortality rates were significantly higher in vancomycin-treated patients than in controls (OR 1.25; 95% CI (1.00, 1.56), p = 0.05) and more precisely than in linezolid (OR 1.29; 95% CI (1.03, 1.62), p = 0.03). Microbiological eradication rates were not statistically different between vancomycin and the comparators (OR, 0.82; 95% CI (0.63, 1.07), p = 0.14). Safety analysis demonstrated comparable adverse event profiles between vancomycin and linezolid for thrombocytopenia, anemia, and hepatotoxicity. Although vancomycin remains a viable option owing to its accessibility and cost-effectiveness, our findings suggest that newer alternatives, particularly linezolid, may offer superior clinical outcomes in specific MRSA infections, especially in cases of pneumonia or high-risk patients. These results have important implications for clinical practice and antimicrobial stewardship programs and support a more nuanced approach to MRSA treatment based on patient-specific factors, infection characteristics, and local resources.

## Introduction and background

Methicillin-resistant* Staphylococcus aureus* (MRSA) is still among the most significant healthcare problems globally and plays a major role in morbidity, mortality, and healthcare costs [1]. This pathogen was first discovered in 1961 as an ever-evolving pathogen primarily associated with healthcare. It was later transformed into an epidemiological entity in the community, presenting specific treatment problems for physicians worldwide [2,3]. In the United States alone, MRSA is responsible for approximately 80,000 invasive infections and 11,000 deaths annually [4,5]. The World Health Organization (WHO) has described MRSA as a "high priority" pathogen, for which there is an urgent need for new antibiotics [6]. This underscores the extent of interest in this pathogen.

MRSA infections present a broad spectrum of clinical manifestations, from benign infections, such as those limited to the skin and soft tissues, to potentially fatal conditions, such as bacteremia and endocarditis [1,7]. The emergence of MRSA strains that are resistant to standard antibiotics has led to the need for the development and testing of alternative treatment options. There has been a seismic shift in therapeutics, with three primary antimicrobials becoming viable treatment options: vancomycin, linezolid, and ceftaroline [1,8].

Vancomycin, a glycopeptide antibiotic, remains the benchmark therapy for severe MRSA infections and has been the mainstay for decades [9,10]. However, the development of vancomycin-intermediate and vancomycin-resistant *S. aureus *strains and doubts regarding vancomycin efficacy in some clinical conditions have initiated interest in newer alternatives [9,10].

Other newer antimicrobial agents developed in recent years include ceftaroline and linezolid, which are effective and promising for treating MRSA infections [11,12]. Ceftaroline is a fifth-generation cephalosporin with a novel anti-MRSA activity by binding more tightly to penicillin-binding protein 2a [12]. Linezolid is an oxazolidinone with alternative activity to protein synthesis inhibitors and a unique benefit of oral bioavailability that supports outpatient therapy options [11,13].

Different components in the literature indicate equivocal comparative effectiveness and safety among these three antimicrobial agents. Liu et al. found an increased probability of linezolid being better associated with the clinical success of MRSA-induced complicated skin and skin structure infections (cSSSIs) than vancomycin [14]. A study has evaluated the effectiveness of ceftaroline in the treatment of complicated MRSA infections, with results indicating favorable outcomes [15]. A previous study compared linezolid and vancomycin in patients with proven MRSA pneumonia and found no significant differences in clinical efficacy [16]. Another study directly compared linezolid and vancomycin in treating MRSA infections and demonstrated that the two treatments were equivalent in efficacy and safety [17]. Additionally, a study evaluated antibiotics for MRSA infections and found that linezolid and tedizolid were more effective in treating MRSA-complicated skin and soft tissue infections. Linezolid is recommended for the treatment of MRSA pneumonia [18]. These findings of past studies highlight the need for an exhaustive comparison of ceftaroline, linezolid, and vancomycin across different types of MRSA infections.

Clinical decision-making in treating MRSA infections is complex and depends on resistance patterns, patient-specific factors, and local epidemiology [19,20]. Although several different treatment options exist, no comparison has been made of the relative efficacy and safety of ceftaroline versus linezolid versus vancomycin, particularly given the potential impact on clinical practice and patient outcomes.

Therefore, this systematic review and meta-analysis assess these three antibiotics. It will provide a firm foundation for appropriate clinical decision-making choices that may positively impact patient outcomes by rationalizing treatment choices. These findings could assist clinicians in making patient-centric, evidence-based decisions and help improve and further develop effective antibiotic stewardship strategies.

## Review

Methods

This systematic review and meta-analysis were conducted in accordance with the Preferred Reporting Items for Systematic Reviews and Meta-Analyses (PRISMA) guidelines.

Study Selection Criteria

The study included adult patients with confirmed MRSA infection who received treatment with ceftaroline, linezolid, or vancomycin, administered either as monotherapy or in combination. Comparator groups included any drug comparable to these three interventional drugs. For outcomes, we primarily examined the clinical cure rate at the time of the cure visit, while secondary outcomes encompassed mortality, microbiological eradication, and adverse events. Randomized controlled trials (RCTs) and observational studies published between 2000 and 2024 were considered for inclusion. Studies were excluded if they were case reports or review articles, focused on non-MRSA infections without subset analysis of MRSA infections, or involved animal subjects. Additionally, studies not published in English or lacking complete outcome data were not included in the analysis.

Search Strategy and Sources

The electronic databases searched included PubMed, Cochrane Central Register of Controlled Trials, Embase, and Web of Science, as well as the references of the included studies and relevant reviews. We developed a comprehensive search strategy for each database using a combination of Medical Subject Headings (MeSH) terms and free-text words. This strategy has been adapted for use with other databases.

Study Selection and Collection

Two independent reviewers screened the titles and abstracts of retrieved records for potential eligibility. The same two reviewers independently assessed the full texts of the potentially eligible studies. Two reviewers independently performed the data extraction using a standardized pre-piloted form. A third reviewer resolved any discrepancies through discussion or arbitration. When necessary, the authors were contacted to obtain the missing data.

Data Items

Data extraction from each study included the study characteristics (first author, year of publication, study design, and sample size), patient characteristics, intervention details, comparator details, and outcomes (clinical cure rate, mortality, microbiological eradication, and adverse events). The risk of bias in the included studies was assessed using the Cochrane Risk of Bias tool for randomized trials (RoB 2).

Results

Search Strategy

**Figure 1:**
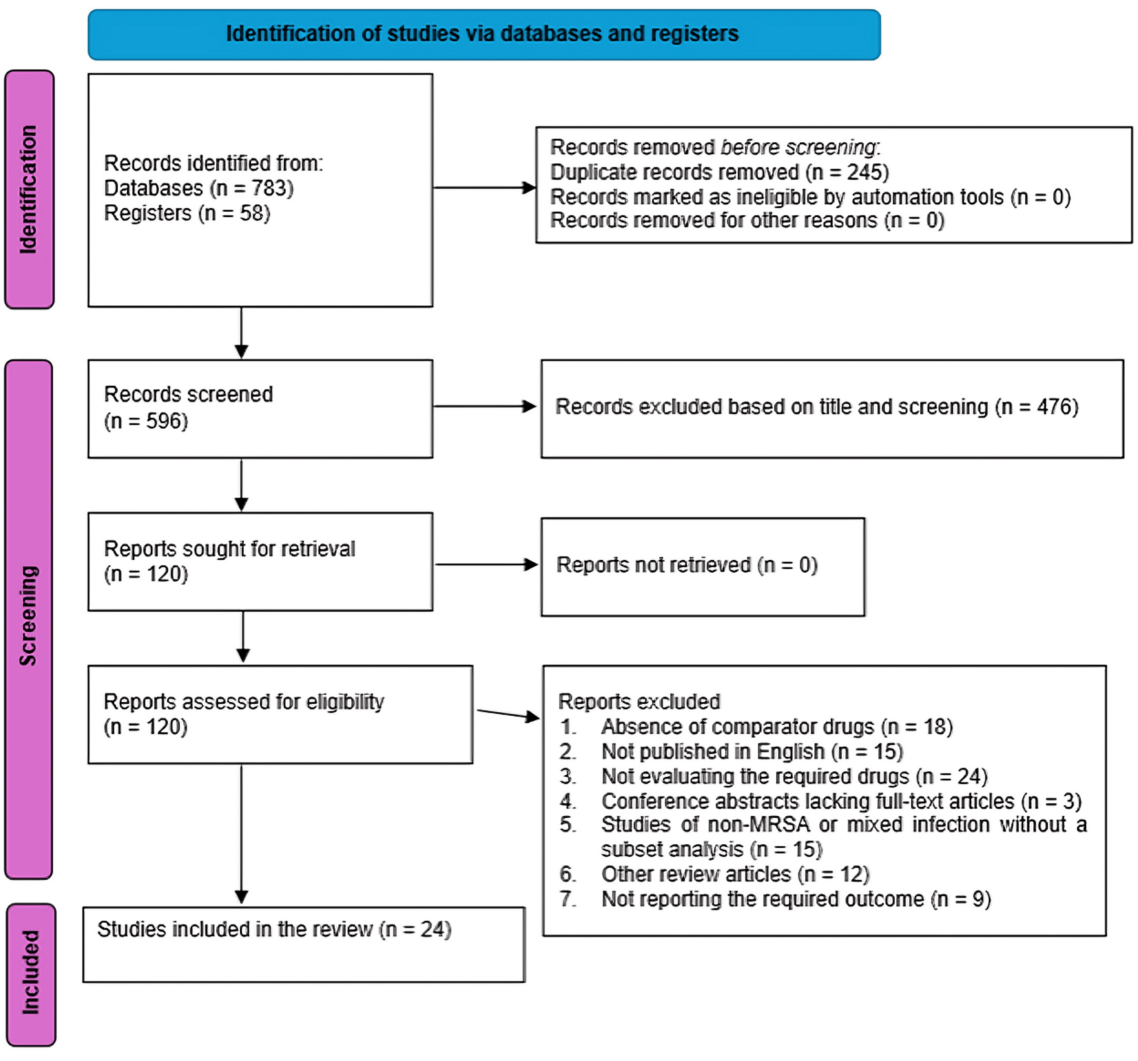
PRISMA diagram summarizing the search strategy PRISMA: Preferred Reporting Items for Systematic Reviews and Meta-Analyses; MRSA: Methicillin-resistant *Staphylococcus aureus*

The electronic search of the different databases led to the retrieval of 841 articles. After duplication assessment, only 596 patients remained. The titles and abstracts of these articles were screened using screening criteria, 476 articles were excluded and 19 reports were not retrieved, resulting in 101 potentially eligible studies. The full articles in these publications were then reviewed. All articles were sought; thus, the eligibility of 101 articles was assessed using a determined criterion. Among these, only 24 met the inclusion criteria. The reasons for exclusion for the other studies included not being published in English (n = 34), not including MRSA (n = 8), not analyzing a subset population of MRSA (n = 7), not including vancomycin, ceftaroline, or linezolid as one of the interventions (n = 24), other secondary studies and reviews (n = 12), conference abstracts lacking full articles (n = 3), lacking comparator drugs (n = 18), and not reporting the required outcomes (n = 9). The search criteria are summarized in the PRISMA diagram (Figure 1).

Characteristics of the Included Studies

This review included 24 studies, of which 17 were RCT, and seven were observational. Different types of MRSA infections include pneumonia, bacteremia, endocarditis, and skin and skin structure infections. The drugs investigated in these studies included vancomycin, linezolid, and ceftaroline. Other drugs included daptomycin, telavancin, and ceftabiprole. All drugs were administered as monotherapy, except vancomycin, which was administered as combination therapy in several studies. The common dosages for the primary drugs included 600 mg 12 hours for linezolid, 600 mg once a day for ceftaroline, and 1000 mg 12 hours for vancomycin (Table 1).

**Table 1:** Characteristics of the included studies NR: Not reported; MRSA: Methicillin-resistant *Staphylococcus aureus*; RCT: Randomized controlled trial

Study	Design	Type of Disease	Drug	Regimen	Sample Size	Mean Age	Vancomycin Trough Levels	Treatment Duration (Days)	Outcomes
Kohno et al., 2007 [17]	RCT	MRSA-related sepsis, pneumonia, and skin and soft tissue infections	Linezolid	600 mg 12 hourly	100	68.4 ± 16.4	NR	10.9 ± 5	Success rate, cure rate, failure rate, and improvement rate
			Vancomycin	1000 mg 12 hourly	51	67.5 ± 16.3	NR	10.6 ± 5.1	
Corey et al., 2010 [21]	RCT	Skin and skin structure infections	Vancomycin plus aztreonam	1000 mg of vancomycin plus 1000 mg of aztreonam	693	48	NR	8 days	Cure rate
			Ceftaroline	600 mg	685	48	NR	8 days	
Rubinstein et al., 2001 [22]	RCT	Nosocomial pneumonia	Vancomycin plus aztreonam	1000 mg of vancomycin plus 1000-2000 mg of aztreonam	193	61.3 ± 18.7	NR	9.6 ± 4.4	Cure rate, failure rate, and success rate
			Linezolid plus aztreonam	600 mg of linezolid plus 100-2000 mg of aztreonam	203	62.8 ± 18.0	NR	8.9 ± 4.4	
Wunderink et al., 2012 [23]	RCT	Nosocomial pneumonia	Vancomycin	600 mg every 12 hourly	176	61.6 ± 17.7	NR	10	Success rate
			Linezolid	Vancomycin 15 mg/kg every 12 hours	172	60.7 ± 18.0	NR	10	
Tong et al., 2016 [24]	Retrospective observational study	Pneumonia	Vancomycin	NR	77	56.6	15-20	NR	Mortality outcomes and success rates
			Linezolid	NR	150	56.5	NR	NR	
Wunderink et al., 2008 [25]	RCT	Ventilator-associated pneumonia	Linezolid	600 mg IV every 12 hours	30	55.7 ± 20.7	NR	NR	Cure rate
			Vancomycin	1000 mg every 12 hours	20	54.9 ± 19.2	NR	NR	
Chavane, 2013 [26]	RCT	Nosocomial pneumonia	Linezolid	600 mg 12 hourly	165	NR	NR	NR	Cure rate
			Vancomycin	15 mg/kg/12 hours	174	NR	NR	NR	
Dennis et al., 2002 [27]	RCT	MRSA infections	Vancomycin	1000 mg twice daily	220	59.8 ± 20.2	12.3 mg/dl	11.3	Cure rate and success rate
			Linezolid	600 mg BID	240	73.33 ± 20.31	NR	12.6	
O’Riordan et al., 2018 [28]	RCT	Acute skin and skin structure infections	Vancomycin plus aztreonam	15 mg/kg/day	427	50.2 ± 16.03	NR	7.0 ± 2.92	Objective response rate, cure rate, and success rate
			Defloxacin	300 mg BID for day 1 to day 12, then 450 mg BID	423	51.2 ± 15.98	NR	7.3 ± 2.97	
Stryjewski et al., 2012 [29]	RCT	Complicated skin and skin structure infections	Vancomycin	1000 mg	102	40 ± 11.3	NR	NR	Clinical cure rate
			TD-1792	2 mg/kg once a day	101	40 ± 11.2	NR	NR	
Pullman et al., 2017 [30]	RCT	Acute skin and skin structure infections	Vancomycin plus aztreonam	Vancomycin 15 mg/kg plus aztreonam plus aztreonam 2g 12 hourly	329	45.3 ± 14.4	NR	6.15 ± 2.62	Objective response, cure rate, and success rate
			Delafloxacin	300 mg 12 hourly	331	46.3 ± 13.91	NR	6.18 ± 2.81	
Overcash et al., 2020 [31]	RCT	Acute bacterial skin and skin structure infections	Vancomycin plus aztreonam	Vancomycin 1000 mg 2-hour infusion plus 1000 mg aztreonam 30-minute infusion every 12 hours	344	50 (20.0-87)	NR	5-10 days	Early clinical response
			Ceftobiprole	500 mg IV infusion over 2 hours every 8 hours	335	51.0 (18-89)	NR	5-10 days	
Rehm et al., 2008 [32]	RCT	MRSA bacteremia and endocarditis	Vancomycin plus gentamicin	Vancomycin 1000 mg every 12 hours, and gentamycin 1 mg/kg every 8 hours	43	54 (25-91)	14.9 mg/L-Vancomycin.	NR	Success rate and safety analysis
			Daptomycin	6 mg/kg every 24 hours	45	57 (22-86)	NR	NR	
Rubinstein et al., 2011 [33]	RCT	Hospital-acquired pneumonia	Vancomycin	1 gram IV every 12 hours	754	63 ± 17.7	NR	NR	Cure rate and safety analysis
			Telavancin	10 mg/kg once a day	749	62 ± 18.5	NR	NR	
Aikawa et al., 2011 [34]	RCT	Skin and soft tissue infections	Vancomycin	70.0 (29-82)	22	70 (29-82)	NR	NR	Clinical and microbiological success rate
			Daptomycin	69.0 (22-92)	88	69 (22-92)	NR	NR	
Itani et al., 2012 [35]	RCT	Complicated skin and skin tissue infections	Linezolid	600 mg q12 hours	95	52.2 ± 19.5	NR	11 (7-17)	Microbiological and clinical success rates
			Vancomycin	15 mg/kg q12 hours	210	49.8 ± 18.4	NR	10 (4-21)	
Kauf et al., 2015 [36]	RCT	-	Vancomycin	As per the site's guidelines (standard protocol)	106	57 ± 53.8	NR	NR	Clinical success rate
			Daptomycin	4 mg/kg OD	118	47.2 ± 54.2	NR	NR	
Wunderink et al., 2003 [37]	RCT	MRSA pneumonia	Vancomycin	1000 mg every 12 hours		61.9	NR	10.7	Clinical cure rate
			Linezolid	600 mg every 12 hours		63.1	NR	11.3	
Kollef et al., 2008 [38]	Retrospective cohort study	MRSA pneumonia	Vancomycin	15 mg/kg every 12 hours	197	57.3	14.9	NR	Mortality outcomes
			Linezolid	600 mg every 12 hours	21	57.3	NR	NR	
Chan et al., 2011 [39]	Retrospective cohort study	MRSA pneumonia	Vancomycin	2272 mg/day	86	53.9	NR	11.2	Mortality outcomes, clinical cure rates
			Linezolid	600 mg every 12 hours	14	56.7	NR	10.6	
Gonzalez et al., 2013 [40]	Retrospective cohort study	MRSA ventilator-associated Pneumonia	Vancomycin	1000 mg every 12 hours	11	46.6	NR	14	Mortality outcomes
			Linezolid	600 mg every 12 hours	17	42.5	NR	14	
Caffrey et al., 2014 [41]	Retrospective cohort study	Hospital-acquired pneumonia	Vancomycin	NR	4493	69.1	NR	NR	Mortality outcomes
			Linezolid	NR	328	69.1	NR	NR	
Peyrani et al., 2014 [42]	Retrospective cohort study	Ventilator-acquired pneumonia	Vancomycin	NR	87	56.0	13	11	Mortality outcomes, clinical cure rate, and success rate
			Linezolid	NR	101	59.0	NR	11	
Takada et al., 2017 [43]	Retrospective cohort study	NR	Vancomycin	1000 mg every 12 hours	17	76.8	15-20	NR	Mortality outcomes
			Linezolid	600 mg every 12 hours	11	77.0	NR	NR	

Quality Assessment of the Observational Study

Most of the included observational studies had good methodological quality. Those with fair quality lacked representativeness of the cohorts because of the small sample population (Table 2).

**Table 2:** The Newcastle-Ottawa scale shows the methodological quality of the included observational studies AHRQ: Agency for Healthcare Research and Quality

Study	Selection	Comparability	Outcome	AHRQ Standard
Kollef et al., 2008 [38]	3	2	3	Good
Chan et al., 2011 [39]	3	2	3	Good
Gonzalez et al., 2013 [40]	2	2	3	Fair
Caffrey et al., 2014 [41]	3	2	3	Good
Peyrani et al., 2014 [42]	3	2	3	Good
Takada et al., 2017 [43]	2	2	3	Fair
Tong et al., 2016 [24]	2	2	3	Fair

**Figure 2:**
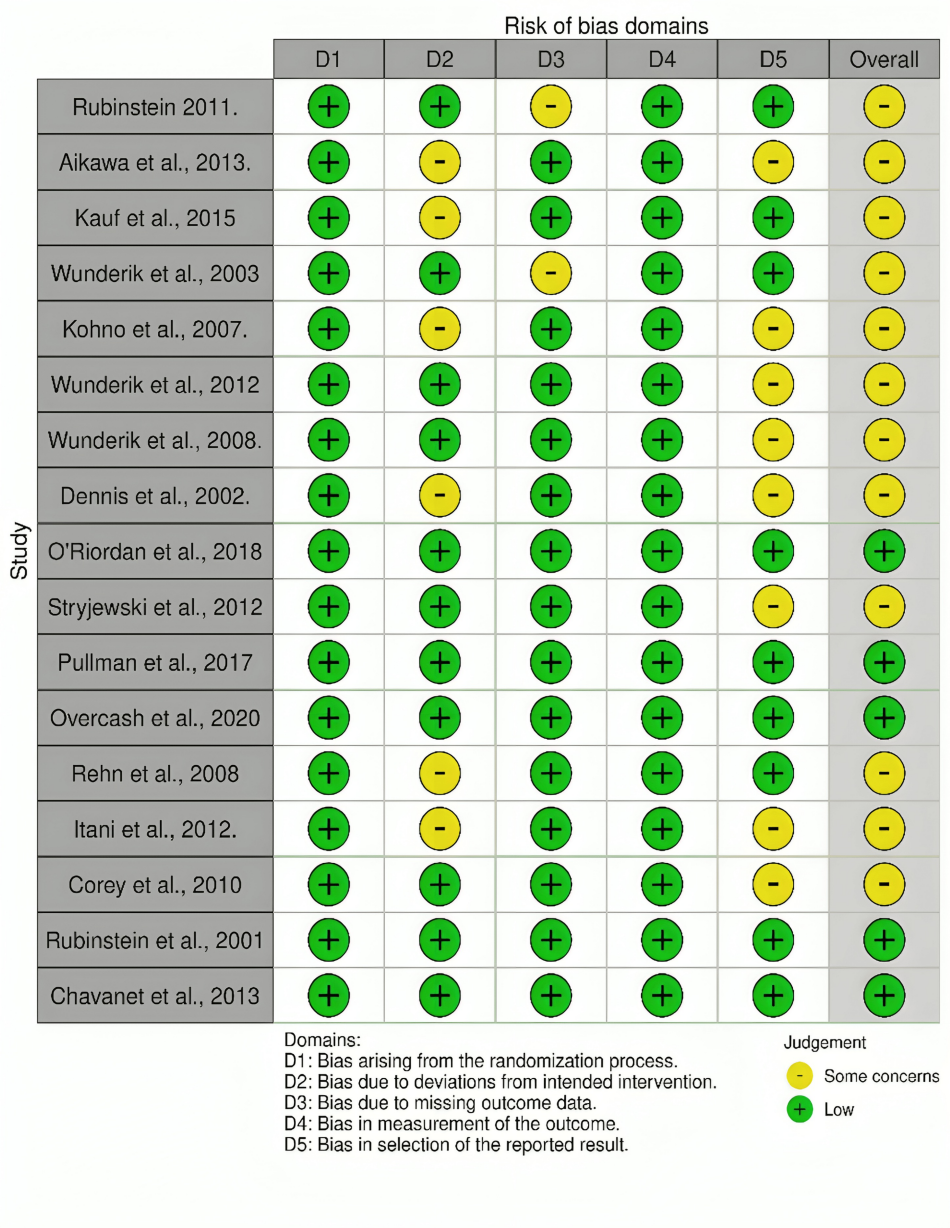
The risk of bias summary indicates the risk of bias in the included randomized controlled trials (RCTs) References: [17,21-23,25-37]

For the RCTs, the most common sources of bias concern were the lack of blinding in the open-label trials and the lack of a published protocol in registries (Figure 2).

Cure Rate

**Figure 3:**
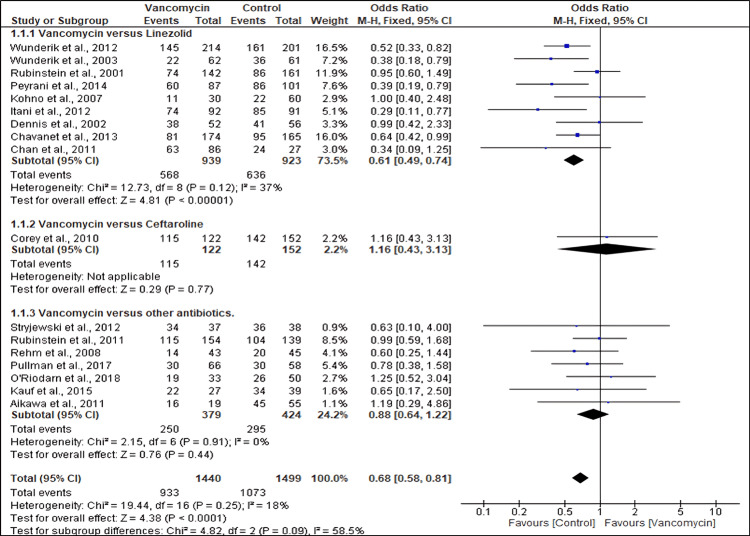
Forest plot showing the cure rates of MRSA patients treated with different antibiotics References: [17,21-23,37,26-30,32-36,42,39] MRSA: Methicillin-resistant *Staphylococcus aureus*

Seventeen studies reported the cure rate of MRSA patients treated with vancomycin compared to the controls. A pooled analysis of the outcomes showed that patients treated with vancomycin had a significantly lower cure rate than the controls (OR: 0.68; 95% CI (0.58, 0.81), p < 0.0001). Similarly, a subgroup analysis showed that the cure rate of MRSA patients treated with vancomycin was lower than that of those treated with linezolid (OR 0.61; 95% CI (0.49, 0.74), p <0.00001). However, no significant difference was observed between patients treated with vancomycin and those treated with ceftaroline and other antibiotics (OR 1.16; 95% CI (0.43, 3.13), p = 0.77) and (OR 0.88; 95% CI (0.64, 1.22), p = 0.44), respectively. There was no significant heterogeneity across studies (I^2^ = 18%, p = 0.25) (Figure 3).

**Figure 4:**
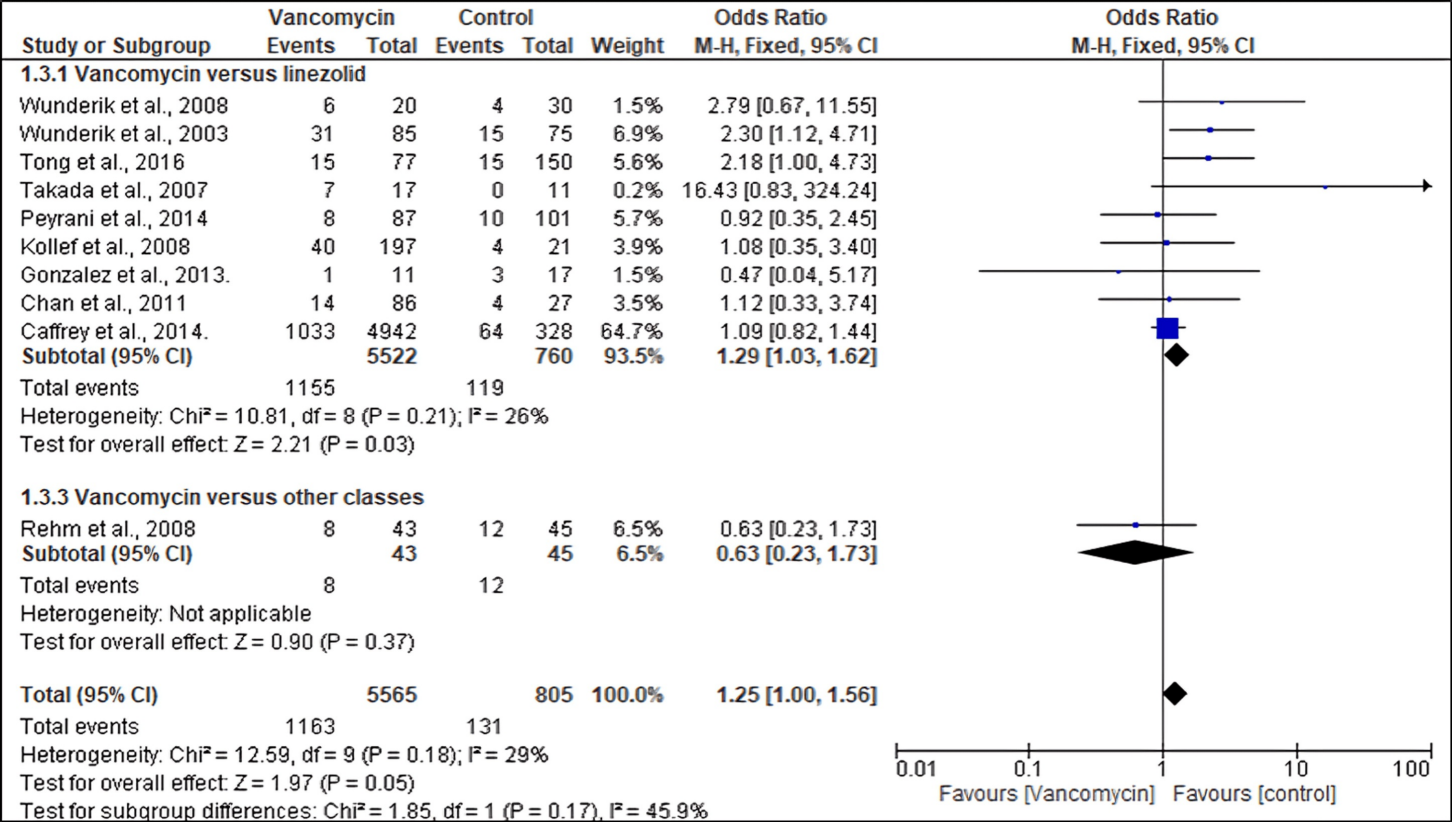
Forest plot showing the mortality rates of MRSA patients treated with different antibiotics References: [25,37,24,43,42,38,40,39,41,32] MRSA: Methicillin-resistant *Staphylococcus aureus*

Eleven studies reported mortality outcomes in patients with MRSA receiving different antibiotics. A pooled analysis of the outcomes showed that the mortality rate was significantly higher in patients treated with vancomycin than in controls (OR, 1.25; 95% CI (1.00, 1.56), p = 0.05). Similarly, subgroup analysis found that the mortality rate was significantly higher in patients treated with vancomycin than those treated with linezolid (OR, 1.29; 95% CI (1.03, 1.62), p = 0.03). However, no significant difference was observed in the mortality of patients treated with vancomycin and those treated with daptomycin (OR, 0.63; 95% CI (0.23, 1.73), p = 0.37) (Figure 4).

Microbiological Eradication Rates

**Figure 5:**
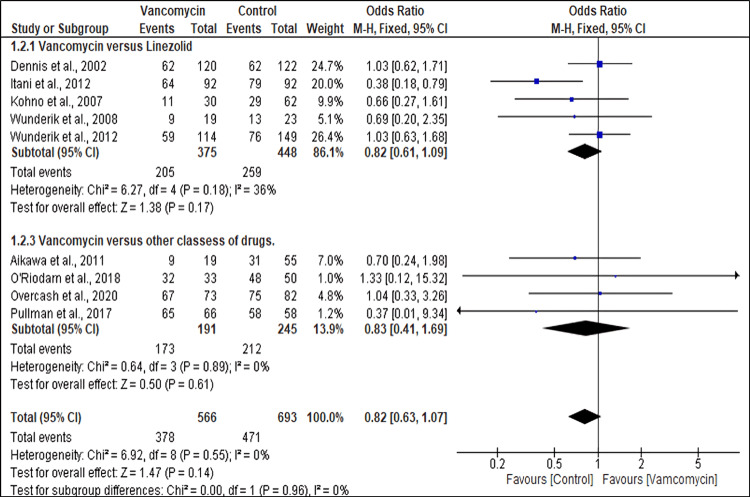
Forest plot showing the microbiological eradication rates of MRSA patients treated with different antibiotics References: [27,35,17,25,23,34,28,31,30] MRSA: Methicillin-resistant *Staphylococcus aureus*

Nine studies reported the outcomes of microbiological eradication rates. The microbiological eradication rates of MRSA extracted from the studies did not significantly differ between the patients who received vancomycin and the controls (OR, 0.82; 95% CI (0.63, 1.07), p = 0.14). Furthermore, a subgroup analysis found no significant difference in the microbiological eradication rates between patients treated with vancomycin and those treated with linezolid (OR 0.82; 95% CI (0.61, 1.09), p = 0.17) or other antibiotics (OR 0.83; 95% CI (0.41, 1.69), p = 0.61) (Figure 5).

Safety Outcomes

**Figure 6:**
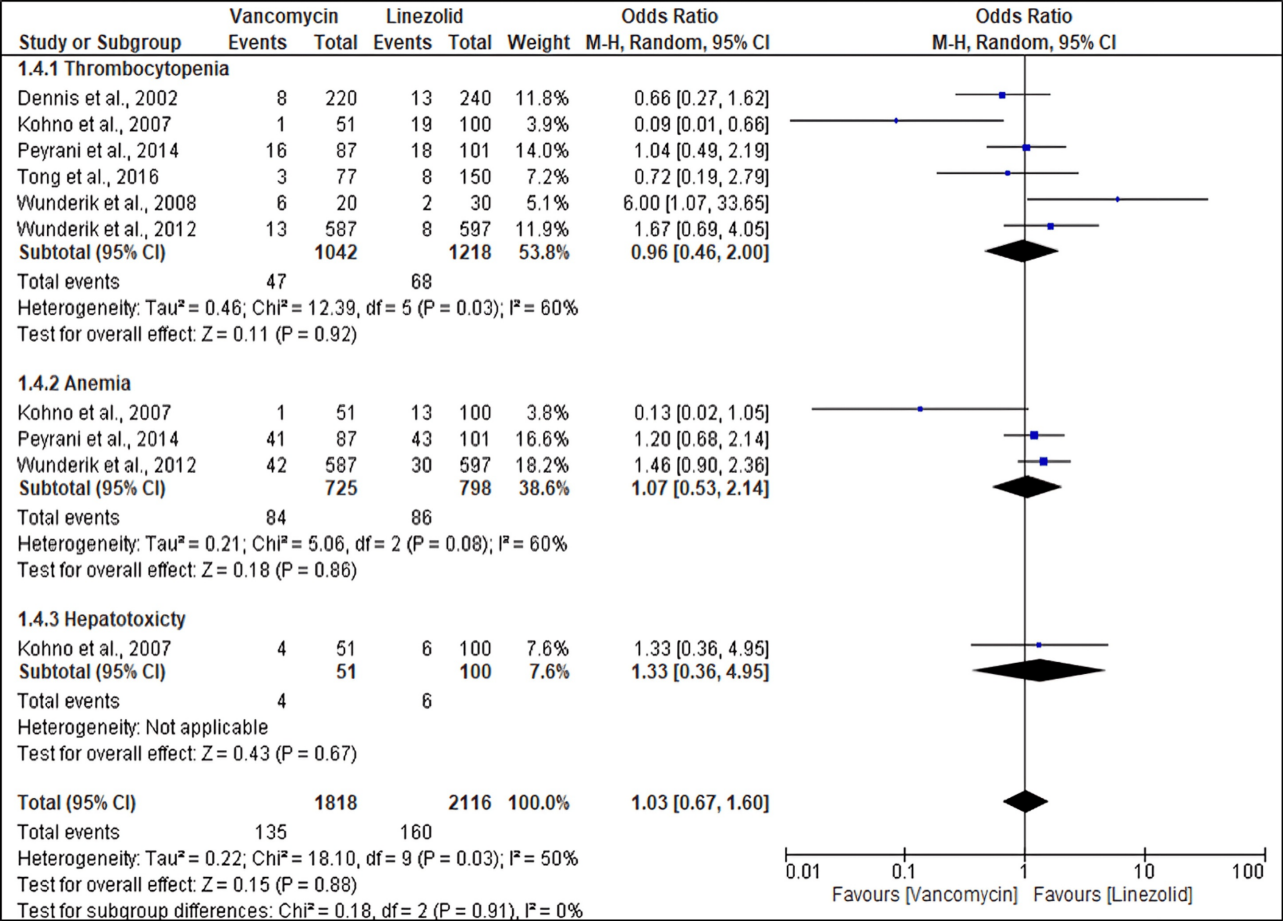
A forest plot showing the incidence of adverse events in MRSA patients treated with vancomycin compared to linezolid References: [27,17,42,24,25,23] MRSA: Methicillin-resistant *Staphylococcus aureus*

This review focuses on the incidence of adverse events in patients with MRSA infections receiving antibiotic therapy. However, in some studies, the outcome of interest was presented as a subset analysis, whereas the safety analysis was presented for the whole population; hence, they were not included in the safety analysis. Furthermore, safety analysis was conducted only on vancomycin versus linezolid because of the limited number of studies. A pooled analysis of data from the studies found no significant differences in the incidence of thrombocytopenia, anemia, hepatotoxicity (OR 0.96; 95% CI (0.46, 2.00), p = 0.92), (OR 1.07; 95% CI (0.53, 2.14), p = 0.86), and hepatotoxicity (OR 1.33; 95% CI (0.67, 1.60), p = 0.67). Moderate heterogeneity was observed across studies (I^2^ = 50%) (Figure 6).

Discussion

This systematic review and meta-analysis provide important comparative insights into the effectiveness of different antibiotics in treating MRSA infections. Our analysis proved that despite being considered the gold standard treatment for MRSA infections, vancomycin showed inferior outcomes compared to newer alternatives, particularly linezolid, in several key metrics. The most important result was the lower cure rate in the vancomycin treatment group than in all other control groups (OR, 0.68; 95% CI (0.58, 0.81)). Moreover, the difference between vancomycin and linezolid was statistically significant (OR 0.61; 95% CI (0.49, 0.74)), which is in line with a previous meta-analysis that reported significantly increased clinical cure rates in patients treated with linezolid compared to vancomycin in both RCTs and cohort studies [44]. Another investigation showed higher clinical success rates at the end of the study for linezolid (57.6%) than for vancomycin (46.6%) in the per-protocol population [23]. In our analysis, no significant differences were observed in the cure rates for vancomycin versus even newer agents such as ceftaroline, indicating that some of the newer options may not offer substantial advantages over vancomycin in terms of clinical cure.

The analysis of mortality outcomes presented a worrying trend, with vancomycin treatment having more than alternative therapies and a relationship with mortality rates (OR 1.25; 95% CI (1.00, 1.56)). This relationship agrees with the findings of previous studies, which raised doubts about the appropriateness of vancomycin in treating severe MRSA infections for the first time [45,46]. The considerably higher mortality rate with vancomycin in contrast to linezolid (OR 1.29; 95% CI (1.03, 1.62)) provides stronger support for the ever-increasing pool of evidence that linezolid may be preferable for some forms of MRSA infection, specifically pneumonia and complicated skin and soft tissue infections. However, one retrospective cohort study of non-critically ill veterans with healthcare-associated pneumonia (HCAP) reported decreased patient mortality associated with linezolid compared with vancomycin [46].

The microbiological eradication rates were not significantly different between the vancomycin and control groups in our analysis (OR, 0.82; 95% CI (0.63, 1.07)), including subgroup analyses that compared vancomycin with linezolid or other antibiotics. In contrast, a network meta-analysis of MRSA skin and soft tissue infections showed that linezolid was superior to vancomycin in improving microbiological success (odds ratio 1.89; 95% CI (1.24-2.86), p = 0.003) [47]. Such inconsistencies could be due to different patient populations, sites of infection, or enhancements in vancomycin dosing strategies over the years.

Our safety analysis revealed analogous profiles of adverse events between vancomycin and linezolid, with no differential incidence noted for thrombocytopenia, anemia, or hepatotoxicity. A retrospective study found that patients receiving linezolid had approximately four times higher incidence of thrombocytopenia than those receiving vancomycin (OR 4.39; 95% CI (2.38-8.08)) [48]. Another study revealed that the adverse effects of linezolid include decreased platelet count, hemoglobin level, and white blood cell count associated with duration-related myelosuppression, including thrombocytopenia, anemia, and leukopenia, which are also associated with elevated liver function tests [49].

A notable strength of our analysis is the inclusion of RCTs and observational studies, which provided efficacy benefits in clinical trials and a more comprehensive view of how these treatments work in the real world. However, the moderate heterogeneity observed in the safety outcomes (I² = 50%) indicates that antimicrobial therapy should be based on individual patient characteristics.

These findings have significant clinical implications, particularly regarding the choice of first-line therapy for MRSA infections. Vancomycin should be kept as the first preference because it is readily available and relatively cheap; however, in specific clinical conditions, such as in some presentations of pneumonia or patients identified as high-risk for treatment failure, the superior cure rates found with linezolid might make the drug preferable.

These results must be interpreted with consideration of the following limitations: First, the included studies differed in their clinical care and treatment success definitions. Second, vancomycin trough levels were infrequently reported in the studies, making it difficult to determine the optimal drug exposure. Third, the meta-analysis did not assess cost considerations likely to influence treatment decisions in practical situations.

Further studies should aim to define which subgroups of patients would benefit the most from newer vancomycin alternatives, considering factors such as the focus on infection, comorbidities, and local resistance rates. Cost-effectiveness research on different therapies, especially in low-resource settings, is warranted to inform evidence-based clinical decision-making.

## Conclusions

This meta-analysis demonstrates that while vancomycin remains a viable treatment option due to its accessibility and cost-effectiveness, newer alternatives, particularly linezolid, show superior outcomes in treating MRSA infections. Specifically, linezolid demonstrated better cure rates and lower mortality rates than vancomycin. However, both drugs showed comparable microbiological eradication rates and safety profiles. These findings suggest that while vancomycin can remain a first-line treatment in resource-limited settings, clinicians should consider linezolid, especially for cases of MRSA pneumonia or high-risk patients. Treatment decisions should be individualized based on patient factors, infection characteristics, local resistance patterns, and resource availability.
